# Does the Same Hyperlactatemia Cut-Off in the Context of Acute Diseases Hold the Same Meaning in Diabetes Mellitus?

**DOI:** 10.7759/cureus.25163

**Published:** 2022-05-20

**Authors:** Inês H Vieira, Maja Petrova, José P Moura

**Affiliations:** 1 Endocrinology, Diabetes and Metabolism, Coimbra Hospital and University Center, Coimbra, PRT; 2 Internal Medicine, Coimbra Hospital and University Center, Coimbra, PRT

**Keywords:** inpatient mortality, metformin-associated lactic acidosis, lactic acidosis, hyperlactatemia, diabetes mellitus

## Abstract

Background

Hyperlactatemia is defined by a lactate concentration of >2 mmol/L, and a lactate concentration of above >4 mmol/L is commonly used to define severe hyperlactatemia. It is a common disorder in critically ill patients and is associated with adverse prognosis. Diabetes mellitus(DM) can also be associated with increased lactate levels at baseline. In this study, we aimed to document the development of severe hyperlactatemia in acute situations among patients with and without DM, to analyze potential contributors to lactate elevation and their impact on mortality, and to analyze whether lactate concentrations of >4 mmol/L have equal prognostic significance in patients with and without DM.

Methodology

A retrospective, cross-sectional study was performed among patients admitted to our internal medicine wards in the context of acute disease with lactate concentrations of ​​≥2 mmol/L. Data were collected regarding age, sex, highest lactate concentrations, cause of hyperlactatemia, DM, and mortality. Statistical analysis was performed using SPSS version 23.

Results

In total, 151 patients with lactate levels of ≥2 mmol/L were analyzed. The mean age of the patients was 78.2 ± 14.9 years, and 55% of the patients were female. Overall, 55.6% of the patients had DM, as well as higher lactatemia of 6.3 ± 3.4 mmol/L (vs 5.1 ± 3.2 in non-DM patients, p = 0.003), with the majority reaching values of >4 mmol/L (vs 34.8% in non-DM patients). When potential contributors to the development of severe hyperlactatemia (lactate >4 mmol/L) were analyzed in DM patients, metformin consumption concomitantly with factors potentiating its accumulation, sepsis/septic shock, ischemia, and neoplasia were the most frequently identified contributors. In non-DM patients, the three former factors were also the most frequently reported. The 30-day mortality rate was 25.82%, with deceased patients also displaying a higher lactatemia of 6.5 ± 3.2 mmol/L (vs. 5.5 ± 3.3 mmol/L in patients who survived) (p = 0.037). In multivariate analysis, lactate values of >4 mmol/L were an independent predictor of mortality in the entire sample and in the subgroup without DM, but not in DM patients.

Conclusions

In our sample, patients with DM had higher lactate levels than non-DM patients. Our analysis raises the possibility that the same lactate values may not have equal capacity to assess prognosis in acute situations in patients with and without DM. Large-scale studies are needed to optimize cut-off points for lactatemia in patients with high baseline values, such as DM patients.

## Introduction

Hyperlactatemia is generally defined as a lactate value of >2 mmol/L. Lactic acidosis is the most common cause of metabolic acidosis in hospitalized patients. Lactate values above 4 mmol/L correspond to more severe hyperlactatemia. Due to the action of acid-base balance adjustment mechanisms, this value can be considered to define lactic acidosis even if there is no effective acidemia (pH <7.35) [1,2]. Bicarbonate of <20 mmol/L and anion gap [3], although generally increased, are within the normal range [1,4].

Blood lactate levels result from a balance between lactate production and elimination. Muscle and brain tissue, red blood cells, skin, and intestine are the main tissues responsible for lactate synthesis [5]. Anaerobic glycolysis is a rapid albeit less efficient way of generating ATP, which increases with stress [3].

In view of a marked increase in lactate production and/or a decrease in its catabolism, hyperlactatemia and eventually lactic acidosis occur [6]. This is a common abnormality in critically ill patients and has an adverse prognostic significance. Lactatemia of >2 mmol/L is an independent predictor of mortality at 30 days, and elevated blood lactate levels are associated with an increase in mortality proportional to lactate values [7]. In patients with sepsis, hyperlactatemia is an independent predictor of mortality [8] and should be used to guide therapeutic measures [9]. Lactate values of >4 mmol/L were associated with longer hospital stays [10] and increased mortality [10,11].

DM has been associated with an increase in lactate levels [12]. Hyperlactatemia can be either a contributor to the development of diabetes or a consequence of the disease. There is evidence that hyperlactatemia can trigger insulin resistance [13]. Excessive adipose tissue is also common in diabetes mellitus (DM) and may contribute to an increase in lactate levels [14]. Lactate has also been explored as a possible biomarker of chronic DM complications, and an association has been suggested between microalbuminuria and diabetic nephropathy [15] and liver dysfunction [16].

Lactate levels appear to play an important role in the central nervous system of DM patients; however, the relationship is complex. The progressive increase in lactate levels appears to be an important pathogenic factor in cognitive impairment associated with DM. During the early stages of the disease, there is an increase in blood glucose, acceleration of glycolysis, and an increase in extracellular lactate that is available to adjacent neurons. In the later stages, due to mitochondrial dysfunction, the use of lactate is reduced leading to its accumulation in the extracellular fluid and causing neuronal dysfunction [17]. It is then possible that the effect of lactate at a central level depends on factors such as the amount, duration, and timing of exposure.

It has also been proposed that hyperlactatemia, in addition to being a common consequence of DM and oncological diseases, contributes to an interaction between the two conditions, leading both to greater insulin resistance and a more malignant phenotype of the neoplasm [18].

The drugs used to treat DM also influence lactate levels, and, in some situations, pioglitazone may be associated with their reduction and metformin with an increase [19].

In the context of acute diseases, DM patients can develop lactic acidosis via several mechanisms. There may be stimulation of the methylglyoxal pathway due to the presence of ketones in diabetic ketoacidosis [5]. In addition, hypovolemia and impaired glucose metabolism present in this acute complication of DM can lead to the accumulation of L-lactate [20].

Metformin inhibits the mitochondrial respiratory chain with the potential to increase lactate genesis. With a half-life of about 6.5 hours, the drug is eliminated unchanged via the kidney [21]. Theoretically, accumulation in view of impaired renal function is expected. Other factors may contribute to the accumulation of this drug and the occurrence of metformin-associated lactic acidosis (MALA), such as hypoxemia, sepsis, shock, alcohol abuse, liver failure, radiological contrast, and ischemic events [22]. However, there are usually several contributing factors at play in patients under metformin and with lactic acidosis, and it is difficult to distinguish in which cases metformin plays a role and in which it is simply a bystander. Currently, it is generally accepted that the risk of MALA is not significantly increased with glomerular filtration rates above 30 mL/minute/1.73 m^2^ [23].

It is important to consider that DM patients are also predisposed to multiple acute complications, which, in turn, can cause hyperlactatemia. For instance, the risk of CV disease makes these patients more prone to hyperlactatemia in the context of heart failure or ischemia. The infectious risk is also increased in these patients, exposing them to the development of sepsis.

While the importance of hyperlactatemia ​​in the prognosis of acute patients, in general, is evident, the magnitude of lactate elevation in DM patients is yet to be analyzed. In a retrospective study with 4,098 patients undergoing cardiac surgery, there was a triple interaction between diabetes, hyperlactatemia, and stress hyperglycemia with mortality. However, a lower prevalence ​​of stress hyperlactatemia was reported in patients with DM, especially in insulin-treated patients [24]. Aleksandar et al., on the other hand, analyzed hyperlactatemia in post-infarction patients and found an association between high lactate levels with worse short and long-term results in DM patients [25].

The fact that DM patients may have elevated baseline lactatemia values for several reasons (described above) raises the question of whether the same cut-off for lactate values holds similar prognostic significance in both DM and non-DM patients.

This study aimed to explore whether a commonly used cut-off value for severe hyperlactatemia/lactic acidosis (lactate >4 mmol/L) had the same prognostic significance (predicting 30-day in-hospital mortality) in patients with and without DM.

## Materials and methods

Study design and patient selection

A retrospective, cross-sectional study was conducted among patients admitted to our hospital’s internal medicine wards over an 18-month period. A computerized search of the digital records of patients admitted and discharged during the study period was carried out at the Internal Medicine Service of our hospital. For our search to be comprehensive, several variants of the Portuguese keywords for “hyperlactatemia,” “lactic acidosis,” and “lactic acidemia” were searched. The records of each of the patients in the initially selected list were evaluated for inclusion and exclusion criteria through an analysis of laboratory data and the record of the emergency episode which led to hospitalization. For prolonged hospitalizations, data available from the last 30 days were analyzed.

Inclusion criteria included patients admitted through the emergency department, patients suffering from acute medical illness, and lactate values of ​​≥2 mmol/L at some time point during the hospital stay. Exclusion criteria included patients who did not satisfy all these criteria, patients who had acute surgery-requiring diseases, and those with insufficient data on essential variables such as lactate values, cause of admittance, and outcome.

Data collection

Information regarding age, sex, index of independence (defined by Katz activities of daily living [26]), the reason for hospitalization, highest recorded lactate values and concomitant pH, probable etiology(ies) of elevated lactate values, history of diabetes, and clinical outcome (death vs. discharge) were collected from electronic records.

The reported lactate and pH values were evaluated using point-of-care blood-gas analyzers. The devices determined lactate using an amperometric method using an enzyme electrode containing lactate oxidase.

When potential contributors to elevated lactate values were not specifically stated, these were defined by careful exploration of electronic records. Because it was essential to the study, all cases where lactate values were not available were excluded. Some patients had missing pH values, in which case the remaining values were analyzed. For the remaining variables, data were generally available.

Data analysis

Statistical analysis was performed using the Statistical Package for the Social Sciences (SPSS) version 23 (IBM Corp., Armonk, NY, USA). For continuous variables, the presence of normal distribution was assessed using the Kolmogorov-Smirnov and Shapiro-Wilk tests. When normal distribution was assumed, an independent sample t-test was used to establish comparisons between the two groups. For most variables, a normal distribution was not assumed, in which case the Mann-Whitney U-test was used to compare two independent groups. The chi-square or Fisher tests were used for group comparison when categorical variables were involved. To evaluate the independent effect of lactate values, multivariate analysis was also performed using logistic regression in the global sample and the subgroups with and without DM.

## Results

In total, 151 patients were included in the study. The selected patients had a mean age of 78.2 ± 14.9 years, with a slight predominance of females (55%). More than half of the individuals in the sample (55.6%) had a diagnosis of DM (previous or made during hospitalization). Hospitalization culminated in death in 25.8% of the cases, with the remaining patients being discharged. Demographic and laboratory data from our sample and comparison between DM and non-DM patients are presented in Table 1.

**Table 1:** Demographic and laboratory characteristics in the global sample and in patients with and without DM. ^1^Except for pH, where n = 144, 81, and 63, respectively. DM: diabetes mellitus; non-DM: patients without diabetes mellitus; SD: standard deviation

	Global sample (n = 151)^1^	DM patients (n = 84)^1^	Non-DM patients (n = 67)^1^	P-value (DM vs. non-DM)
Age, years (mean ± SD)	78.2 ± 14.9	79.9	76.2	0.716
Gender (%female/%male)	55.0/45.0	58.3/41.7	50.7/49.3	0.352
Katz score (number)				0.878
0	69	39	31	
1	10	7	3	
2	11	6	5	
3	5	4	1	
4	9	4	5	
5	2	1	24	
6	45	24	21	
pH (mean ± SD)	7.4 ± 0.1	7.4 ± 0.2	7.4 ± 0.1	0.186
Lactate levels, mmol/L (mean ± SD)	5.8 ± 3.3	6.3 ± 3.4	5.1 ± 3.2	0.008
Lactate >4 mmol/L (%)	60.9	71.4	47.8	0.003
Deceased (%)	25.8	25.0	26.9	0.853

The groups of patients with and without DM were homogeneous regarding age distribution, with a mean age of 79.9 ± 11.3 years in patients with DM and 76.2 ± 18.3 years in the remainder (p = 0.716). There was also no statistically significant difference in the distribution by sex (p = 0.352) or regarding dependence index (p = 0.878).

**Figure 1:**
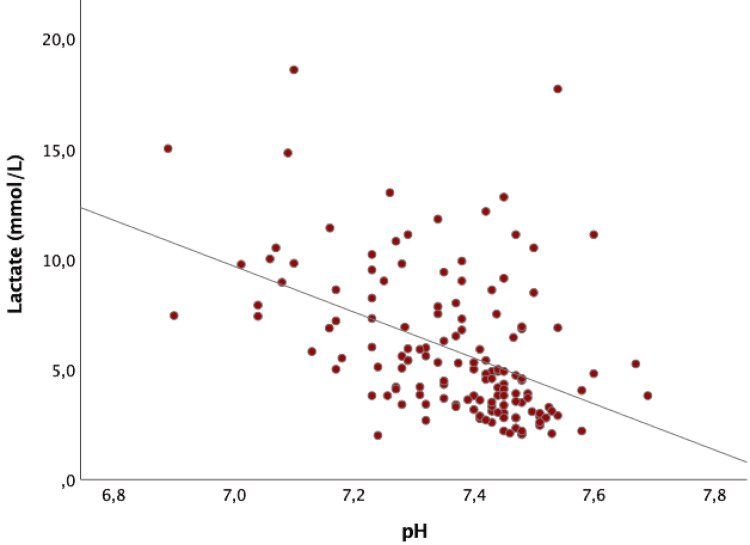
Correlation between lactate and pH values. A moderate, negative correlation between lactate and pH values was found (Spearman test, rs = -4.89; p < 0.001).

In the global sample, the mean lactate levels were 5.8 ± 3.3 mmol/L. pH values were available for 144 individuals, with mean values of 7.4 ± 0.1. There was a moderate, negative correlation between lactate and pH values (Spearman test, rs = -4.89; p < 0.001). Lactate values of >4 mmol/L were significantly associated with a pH of <7.35 (chi-square test, p < 0.001) (Figure 1).

Lactate values ​​were higher in patients with DM who had a mean value of 6.3 ± 3.4 versus 5.1±3.2 for individuals without DM (p = 0.008). The majority (71.4%) of patients with DM had lactate values of ​​>4 mmol/L, while only 47.8% of patients without DM registered this range of lactate values, with a statistically significant difference between groups (p = 0.003).

Potential contributors to severe hyperlactatemia/lactic acidosis (here defined by lactate of >4 mmol/L) were evaluated in each of the 92 patients who reached this value, with more than one factor found in some patients. In the subgroup with DM and lactate of >4 mmol/L (N = 60), the most frequently reported potential contributors included taking metformin simultaneously with factors likely to enhance its accumulation (n = 37), sepsis/septic shock (n = 24), ischemia (n = 7), and presence of neoplasia (n = 5). In the subgroup of patients without DM and lactate of >4 mmol/L (N = 32), sepsis/septic shock (n = 6), ischemia (n = 6), and neoplasia (n=4) were frequently recorded. Table 2 and Table 3 detail all the contributing factors identified for patients with and without diabetes, respectively.

**Table 2:** Potential contributors for severe hyperlactatemia/lactic acidosis in diabetes mellitus patients. In some patients, more than one factor was identified.

	n	% (in 60 patients)
Metformin	37	61.7
Sepsis/septic shock	24	40.0
Ischemia	7	11.7
Neoplasm	5	8.3
Liver failure	4	6.7
Decompensated heart failure	3	5.0
Non-septic shock	3	5.0
Cardiorespiratory arrest	1	1.7
Diabetic ketoacidosis	1	1.7
Severe anemia	1	1.7

**Table 3:** Potential contributors for severe hyperlactatemia/lactic acidosis in patients without diabetes mellitus. In some patients, more than one factor was identified.

	n	% (in 32 patients)
Sepsis/septic shock	16	50.0
Ischemia	6	18.8
Neoplasm	4	12.5
Intoxication	3	9.4
Decompensated heart failure	2	6.2
Liver failure	2	6.2
Non-septic shock	2	6.2
Convulsive crisis	1	3.1
Innate metabolism disease	1	3.1
Hypoxia	1	3.1
Cardiorespiratory arrest	1	3.1

In the entire sample of 151 patients, those who died reached higher lactate values ​​of 6.5 ± 3.2 (vs. 5.5±3.3 mmol/L in the remaining patients), which was statistically significant (p = 0.037). There was also a tendency toward this association when analyzing patients with DM and without DM separately. DM patients who died had lactate values of 7.4 ± 3.6 mmol/L, which were significantly higher than those of DM patients who survived (average lactate of 5.9 ± 3.3 mmol/L) (p = 0.062). For those without DM who died, mean lactate values were 5.4 ± 2.5 vs. 5.0 ± 3.4 in the remaining non-DM patients (p = 0.101).

**Figure 2:**
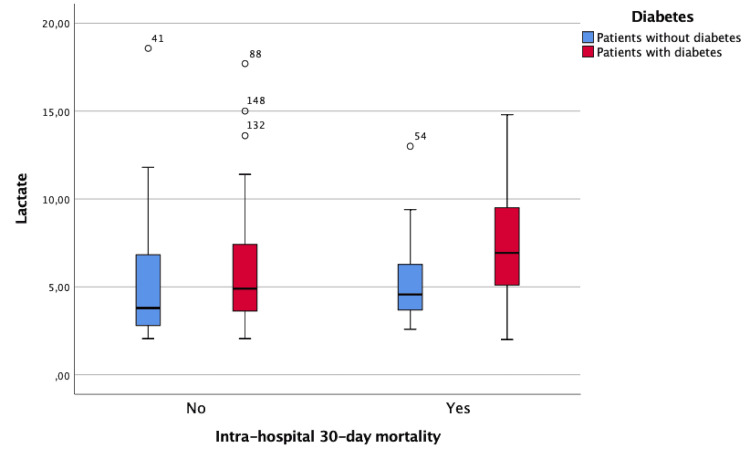
Lactate values in patients with and without diabetes clustered by outcome (intra-hospital 30-day mortality).

The outcome of death was more frequent in patients with lactate values of >4 mmol/L than in those who did not reach this value (odds ratio = 2.26; p = 0.046), and, as previously stated, DM patients had more frequent lactate values of >4 mmol/L. However, lactate values of patients with DM tended to be higher than those of the remaining patients, both in those who survived (5.9 ± 3.3 vs. 5.0 ± 3.4, p = 0.018) and who died during the hospital stay (7.4 ± 3.6 vs. 5.4 ± 2.5, p = 0.069) (Figure 2).

Logistic regression was performed to assess the impact of age, gender, presence of lactic acidosis, and diabetes on mortality. In general, the model was statistically significant (p = 0.030) and explanatory for 6.9-10.1% of the probability of death. Age and lactate of >4 mmol/L were independent predictors of mortality with odds ratios of 1.037 and 2.705, respectively (Table 4).

**Table 4:** Influence of age, sex, diabetes mellitus, and presence of lactic acidosis in intra-hospital mortality. SE: standard error

	B	SE	Wald	P-value
Age	0.037	0.016	4.967	0.026
Sex	-0.292	0.404	0.522	0.470
Diabetes mellitus	-0.428	0.408	1.097	0.295
Lactate >4 mmol/L	0.995	0.444	5.024	0.025

In the analysis of subgroups of patients with and without DM, the presence of lactic acidosis was only a predictor of mortality in patients without diabetes with an odds ratio of 3.953 (p = 0.027). In patients with diabetes, a lactate value of >4 mmol/L was not an independent predictor of mortality during hospital stay (p = 0.520) (Table 5).

**Table 5:** Influence of age, sex, and presence of lactic acidosis in intra-hospital mortality in patients with and without diabetes mellitus. SE: standard error; DM: diabetes mellitus

		B	SE	Wald	P-value
Age	Non-DM	-0.046	0.023	3.965	0.046
	DM	0.033	0.026	0.840	0.206
Sex	Non-DM	-0.717	0.639	1.256	0.262
	DM	0.019	0.538	0.001	0.971
Lactate >4 mmol/L	Non-DM	1.375	0.622	4.887	0.027
	DM	0.575	0.627	0.840	0.359

## Discussion

Hyperlactatemia is a common disorder in critically ill patients and has an adverse prognostic significance. It is used as a marker of the need for aggressive therapeutic measures [9].

Previous data suggest that patients with DM tend to have higher lactate levels due to factors inherent to their metabolism [13], the frequent presence of obesity [14], and the contribution of drugs [19]. It is conceivable that the relative basal hyperlactatemia of patients with DM interferes with the interpretation of lactate levels in an acute context.

Our sample, which was selected based on lactate levels, encompassed a high percentage of DM patients (56%), significantly higher than the estimated prevalence of DM in the Portuguese population above 65 years of age which is estimated to be approximately 23.8% [27]. Patients with DM are predisposed to several acute complications, commonly associated with hyperlactatemia, which may in part explain why these patients constituted such a significant part of the sample.

Patients with DM had higher lactate values ​​than those without DM. These data suggest that DM is a risk factor for more elevated lactate values even in an acute setting. A significant number of our DM patients had metformin as a possible contributing factor to their hyperlactatemia in the setting of acute kidney injury. However, in the absence of measurements of metformin blood values, it is not possible to conclude in which patients it contributed to the increase in lactate values. Posma et al. reported higher lactate values at admission in the intensive care unit in patients taking metformin versus non-users, which were even higher in those with more severe kidney injury. However, the differences disappeared within 24 hours. The authors highlight the importance of being aware of the possible influence of metformin use in this biomarker [28].

Lactate values above 4 mmol/L were more frequently associated with mortality and these values were observed more often in patients with DM. However, the presence of higher lactate values in DM patients was registered not only in those who died but also in patients who survived.

When our entire sample was analyzed, a prognostic significance of hyperlactatemia was noted, with the highest lactate values ​​present in patients who died. This is in agreement with previous data, which show a correlation between hyperlactatemia and mortality and increased mortality [7,10,11].

In multivariate analysis, the presence of lactatemia (>4 mmol/L) was an independent predictor of mortality in the global sample and the subgroup of patients without DM, but not in DM patients. Given these data, it is hypothesized that patients with DM had higher baseline lactate values, as suggested by previous studies [13,14,19]. Interpretation of hyperlactatemia in these patients is challenging for predicting prognosis in an acute context.

There are few data specifically evaluating the differences in the capacity of predicting mortality of traditionally used lactate cut-off points in DM versus non-DM patients. In a group of patients with acute myocardial Infarction, Aleksandar et al. also found higher serum lactate in DM patients versus non-DM patients. According to the authors, in DM patients, high lactate levels were associated with a higher risk of negative outcomes compared with non-DM patients. Conversely, they reported that while lactate >5 mmol/L was associated with a lethal outcome in all non-DM patients, the same was not true for DM patients [25]. Although based on low case numbers, this last finding is similar to that in our study.

Our study has some limitations, such as its retrospective design and the fact that it was based on clinical records. The sample size was also a limiting factor, not allowing analysis by a group of acute diseases and functional status.

Our data suggest that DM is associated with higher lactate values in the setting of acute illnesses and that the same lactate cut-off values may not be equivalent concerning prognostic significance in patients with and without DM. However, we would like to highlight that hyperlactatemia in a critical patient should not be disregarded, irrespective of the presence or absence of DM. However, it is important to be aware that DM itself, as well as the drugs used to treat it and the complications associated with the disease, may contribute to further elevating this biomarker.

In the future, it would be useful to conduct large-scale studies with analysis of lactate values ​​in patients with and without DM with different types of acute diseases. This type of investigation would have the potential to certify (or even redefine) lactate-specific cut-off points ​​for patients with DM in specific who are particularly vulnerable to several acute complications.

## Conclusions

Several data indicate that DM can be associated with higher baseline lactate values ​​compared to individuals without DM. In the setting of acute diseases, lactate values are considered an important predictor of mortality. Our data suggest that this biomarker is even more elevated in DM patients in the context of acute illnesses when compared with critical patients without diabetes. As such, commonly used cut-off points for hyperlactatemia during critical care may not have an equivalent prognostic significance in people with and without DM. Hyperlactatemia in a patient with the acute disease should never be disregarded; however, larger-scale studies are needed to better define the influence of DM, its associated complications, and therapies in lactate values in the setting of acute diseases.
